# Apical periodontitis in southern Estonian population: prevalence and associations with quality of root canal fillings and coronal restorations

**DOI:** 10.1186/s12903-017-0429-7

**Published:** 2017-12-12

**Authors:** Veiko Vengerfeldt, Reet Mändar, Minh Son Nguyen, Silvia Saukas, Mare Saag

**Affiliations:** 1 Institute of Dentistry, Faculty of Medicine, University of Tartu, Raekoja plats 6, 51003 Tartu, Estonia; 2 Institute of Biomedicine and Translational Medicine, Faculty of Medicine, University of Tartu, Ravila 19, 50411 Tartu, Estonia; 3 Competence Centre on Health Technologies, Tiigi 61b, 50410 Tartu, Estonia; 4 Department of Prosthodontic, Danang University of Medical Technology and Pharmacy, 99 Hung Vuong, Da Nang, 550000 Vietnam

**Keywords:** Apical periodontitis, Epidemiology, Quality of root canal filling, Quality of restoration

## Abstract

**Background:**

Apical periodontitis (AP) is an inflammatory disease around the apex of a tooth root. Reported prevalence of AP ranges from 27% to 83% while the data about most post-Soviet countries are still missing. Knowing the prevalence of AP within a certain population helps to plan the treatment need and evaluate the success of endodontic interventions. We aimed to collect data about prevalence and determining factors of AP for the first time in Estonia.

**Methods:**

The cross-sectional study included 6552 patients (age 35.5±19.2 years). Radiographic examination was applied to investigate the prevalence of AP and quality of endodontic treatment.

**Results:**

AP was diagnosed in 54.7% of subjects. Endodontically treated teeth were present in 58.2% of subjects. Periapical finding was present in 44.6% of endodontically treated and in 30.8% of untreated teeth.

Out of 181,495 teeth, 52.7% were intact. AP was diagnosed in 6.3% of teeth, 6.9% of teeth were endodontically treated.

Risk factors for AP included caries (OR = 2.30), male gender (OR = 1.44), too short (OR = 1.76) or too long root canal filling (OR = 2.51), root canal filling of low density (OR = 1.61) while not orthodontic appliance. Lower AP risk was associated with restoration of the tooth – filling (OR = 0.45), crown (OR = 0.34) and bridge (OR = 0.33).

**Conclusions:**

AP is highly prevalent in Southern Estonian population. Most cases are associated with root canal-treated teeth. The overall quality of root canal fillings tends to be low, consistent with the mediocre outcome of treatment. Considerable efforts are required to improve the standards of endodontic treatment.

**Electronic supplementary material:**

The online version of this article (10.1186/s12903-017-0429-7) contains supplementary material, which is available to authorized users.

## Background

Apical periodontitis (AP) is an inflammatory disease around the apex of a tooth root that is caused by infection in root canal system [1, 2]. Chronic AP is characterized by local inflammation in response to the infection, periapical bone destruction and degradation of extracellular matrix [3]. The state and progression of the disease are greatly influenced by the interactions of various inflammatory and anti-inflammatory molecules during the host defense reaction [3, 4]. There is evidence suggesting increased level of systemic inflammation in patients with AP [5]. Furthermore, systemic conditions i.e. poor glycemic control [6], diabetes [7] viral diseases [8] and lowered bone mineral density [9] seem to predispose to the occurrence of AP. Different epidemiological cross-sectional studies show AP being a widespread condition in many countries, and its prevalence is positively correlated with age (Additional file 1: Table S1), gender and socioeconomic status of the region. Reported prevalence of AP ranges from 27% in Finland up to 83% in Jordan [10, 11]. However data about most post-Soviet countries are still missing, being present only for Lithuania, Latvia and Belarus [12–14].

Many studies have shown root canal treatment as one of the biggest risk factors for AP as the latter is always more frequent in treated than non-treated teeth **(**Additional file 1: Table S2). At the same time several studies have indicated that AP is closely related to the quality of dental treatment. Because of that, the determination of the treatment quality alongside the prevalence of AP is utmost important. Knowing the distribution and prevalence of AP within a certain population helps to plan the treatment need and evaluate the success of endodontic interventions [15, 16].

The aim of this study was to collect data about the prevalence and determining factors of apical periodontitis for the first time in Estonia.

## Methods

### Study population

In Estonia, oral health services for adults are available only in private sector. Only emergency care like abscess incision with drainage and acute extraction is supported by the public sector under the Ministry of Health via the Estonian Health Insurance Fund. The private sector has also the main responsibility to provide services for children and adolescents, free-of-charge up to 19 years of age supported by public sector under Ministry of Health.

The sample for this cross-sectional study consisted of 6552 patients, which is 0.5% of the total Estonian population and about 7% of the population of Tartu. Mean age of the study group was 35.5 ± 19.2 years, age ranging from 3 to 93 years. All the orthopantomogram (OPTG) X-rays were taken for the first time at the radiology department at the Clinic of Dentistry, Tartu University Hospital, Estonia between November 2010 and May 2012 only for the consultation and/or treatment purposes.

### Ethics statement

The study was conducted in compliance with the "Ethical principles for medical research involving human subjects" of Helsinki Declaration and approved by Ethics Review Committee (ERC) on Human Research of the University of Tartu (protocol no: 246/T-19). All of the collected data were coded and isolated; personal data and measurements data were kept separately.

### Radiographic examination

All radiographs were carried out by the same experienced dental radiologist using a panoramic digital radiography device Planmeca ProMax 3D Plus (*Planmeca Oy, Helsinki, Finland)* with imaging values between 54 and 96 kV and 1–14 mA, depending on the subject’s size. For assessment of the radiographs, a HP 22uh 21.5-in. LED Backlit Monitor 1920 × 1080 @ 60 Hz using Romexis Imaging Software *(Planmeca Oy, Helsinki, Finland)* was used. For better assessment the observers were able to use all software image enhancement functions whenever they felt the necessity for it.

At first one specialist (V.V.) in the Department of Oral and Dental Diseases examined all radiographs. All teeth were recorded according to FDI nomenclature, in all teeth the variables and findings listed in Additional file 1: Table S6 were assessed. To identify teeth with AP only the presence or absence of periapical radiolucency was used [17]. The root with the lowest quality of treatment was included into study in case of multirooted teeth. Thereafter the second observer (S.S.) in department of oral and dental diseases examined all radiographs. She also filled all the variables for all the teeth in all radiographs the same manner as first observer (V.V). In cases of disagreement the third observer (M.S.) resolved the discrepancy.

### Calibration of observers

Calibration of observers was carried out on a selected set of 104 orthopantomographs from an unpublished pilot study from the same investigation group. The interobserver agreement scores gave Kappa-value of 0.51 for presence of periradicular radiolucency, 0.55 for length of root canal filling (RCF), 0.44 for quality of lateral seal, 0.56 for quality of coronal fillings, 0.63 for quality of crowns. Because the interobserver agreement was moderate (0.44–0.63), a highly experienced dentist served as the third observer (M.S.) for cases where disagreement occurred.

## Results

### Patients

In this study, 6552 subjects ranging in age from 3 to 93 years (mean 35.5 ± 19.2 years) were assessed, of them 2563 (39.1%) were male and 3989 (60.9%) were female. Majority of the subjects (62.6%) were 10 to 44 years old and the biggest age-group (11.3%) included the patients in the age of 15–19 years (Table 1 and Additional file 1: Table S3). Only 209 subjects out of 6552 had completely intact teeth.

**Table 1:** Distribution of patients with AP and/or root canal filling (RCF) by gender and age

Variable	Total patients		pAP patient		*p*-value	Patients with RCF		*p*-value	Patients with RCF teeth with AP (sAP)		*p*-value
	No. of patients	%^a^	No. of patients	%		No. of patients	%^a^		No. of patients	%^b^	
Gender											
Female	3989	60.9	1152	28.9	<0.001	2435	61.0	<0.001	1855	76.2	0.487
Male	2563	39.1	869	33.9		1380	53.8		1065	77.2	
Age group											
<10	361	5.5	4	1.1	<0.001	8	2.2	<0.001	6	75.0	<0.001
10–19	1326	20.2	103	7.8		220	16.6		120	54.5	
20–29	1295	19.8	295	22.8		640	49.4		431	67.3	
30–39	1017	15.5	381	37.5		768	75.5		571	74.3	
40–49	890	13.6	410	46.1		766	86.1		634	82.8	
50–59	779	11.9	362	46.5		690	88.6		573	83.0	
60–69	512	7.8	272	53.1		436	85.2		356	81.7	
70+	372	5.7	194	52.2		287	77.2		229	79.8	
Total	6552	100	2021	30.8		3815	58.2		2920	76.5	

Chi-Square test was applied

^a^Per cent from total number of subjects was calculated

^b^Per cent from patients with root canal fillings was calculated

**Fig. 1:**
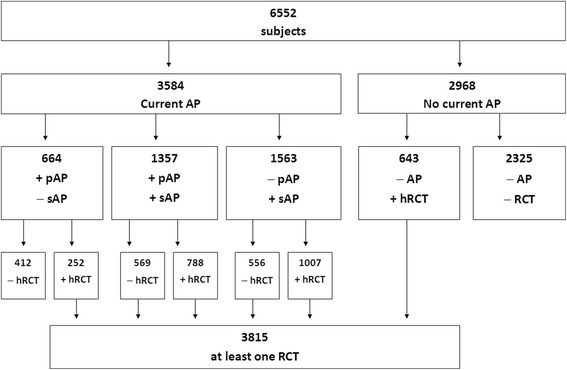
Overview of the study subjects. Altogether 3584 subjects with AP were investigated (of them 2021 had pAP at least in one tooth and 2920 had sAP at least in one tooth) as well as 2968 subjects without current AP. Legend: AP, apical periodontitis; pAP, primary AP; sAP, secondary AP; RCT, root canal treatment; hRCT, healthy RCT in other teeth; +, present; −, absent

Of the total sample, 54.7% (3584 patients) had AP, and 58.2% (3815 patients) had root canal treatment (RCT) while 2737 patients (41.8%) had no RCT visible on X-rays (Fig. 1). The age range in subjects with AP and/or RCT was from 5 to 90 years (mean 44.5 ± 16.5 years).

Periapical finding in endodontically treated teeth (secondary apical periodontitis, sAP) was present in 2920 subjects (76.5% of subjects with previous RCT, 44,6% of total subjects), whereas 2021 patients (30.8% of total subjects) had AP in non-endodontically treated teeth (primary apical periodontitis, pAP) (Fig. 1).

Radiolucencies were observed most often in the mandibular first molars (in 26.7% of patients) and least often in the mandibular canines (0.4%) and mandibular central and lateral incisors (0.8%).

Table 1 shows the distribution of AP and RCT by age groups and gender. Prevalence of females having RCT was significantly higher than those of males, but males had more pAP. There was no difference between males and females in term of sAP. The proportion of patients with pAP, sAP and RCT increased by age (*p* < 0.001), this shift was more prominent between the ages of twenties and thirties (Table 1).

### Teeth

The 6552 patients had altogether 181,495 teeth (Additional file 1: Tables S4 and S5) and the mean number of teeth per patient was 27.7 ± 2.3.

Out of 181,495 teeth, 52.7% were intact while 47.3% of teeth were impacted by caries, restoration, endodontic treatment, and/or AP. There were 36,452 teeth with visible caries (primary or secondary type or both; at the same time the carious tooth could have any type of restoration, AP and/or endodontic treatment) and 49,303 teeth that had any type of restoration (filling, crown, bridge abutment etc). Overview of the status of the teeth is presented in Table 2.

**Table 2:** Overview of dental health in the 6552 study subjects

Condition	Number of teeth involved per patient Mean ± SD
Number of intact teeth	14.6 ±11.7
Number of non-intact teeth^a^	13.1 ± 8.3
Number of teeth with caries^b^	5.6 ± 5.1
Primary caries	1.8 ± 2.6
Secondary caries^c^	3.8 ± 4.1
Number of teeth with restoration^d^	9.1 ± 6.5
Teeth with fillings	6.9 ± 5.7
Crowned teeth	0.3 ±1.0
Bride abutment teeth	0.3 ± 1.1
Number of teeth missing^e^	2.8 ± 4.6

^a^Including any type of restoration, any type of caries, any type of endodontic treatment, and/or apical periodontitis

^b^The caries can be primary or secondary type or both; on the same time the carious tooth can have any type of restoration, apical periodontitis and/or endodontic treatment

^c^If the patient had separately both types of caries (primary and secondary in one tooth together) they were grouped in secondary caries group

^d^Filling, crown, bridge abutment etc

^e^Not including wisdom teeth

Among 181,495 teeth, AP was diagnosed in 11,438 teeth (4898 pAP and 6540 sAP), while 12,605 teeth (8498 belonged to female and 4107 to male patients) were endodontically treated.

The highest prevalence of pAP was seen in lower molars (6.6%), followed by upper molars (3.0%) and lower premolars (2.5%). The highest prevalence of sAP was seen also in lower molars, followed by lower incisors and lower premolars (Additional file 1: Table S4). The most RCT teeth were the right mandibular first molars (19.9%), followed by the left mandibular first molars (19.4%) and the right maxillary first molars (15.2%). The least treated teeth were the left mandibular lateral incisors (1.0%) closely preceded by right mandibular central and lateral incisors (1.1% both) (Additional file 1: Table S5).

There was a 2.30 times higher risk for AP for the teeth of mandible than that of maxilla. Molars had 1.79 times higher risk for AP in comparison with incisors. Teeth with caries had 2.30 times higher risk for AP compared to teeth without caries. In addition, male gender was associated with risk for AP (1.44 times). At the same time the teeth with orthodontic appliance did not have higher risk for AP (Table 4).

### Quality of treatment

Out of the 12,605 endodontically treated teeth, 11,956 teeth (94.8%) were treated using pulpectomy and 649 teeth (5.2%) were treated using the pulpotomy method (Table 3). Of 11,956 pulpectomy teeth, 48.6% teeth were without AP while of 649 pulpotomy teeth, only 13.5% teeth were without AP. In total, 51.9% of endodontically treated teeth presented with AP.

**Table 3:** Quality of treatment

Assessed quality	Value	Number of teeth		
		Healthy	With AP	Total
Endodontically treated teeth		48.1%	51.9%	12,605
Length of root canal filling (*n* = 11,956)^a^	Less than 2 mm from apex	60.9%	39.1%	3708
	More than 2 mm from apex	43.5%	56.5%	7546
	Over apex	38.5%	61.5%	702
Root canal filling density (*n* = 11,956)^a^	Dense	58.1%	41.9%	2870
	Inhomogenous	41.4%	58.6%	9086
Type of restoration (*n* = 12,605)^a^	No restoration	29.3%	70.7%	915
	Filling	48.3%	51.7%	9525
	Crown	54.4%	45.6%	1361
	Bridge retainer	55.8%	44.2%	804
Quality of prosthetic restoration (*n* = 2165)	Adequate	59.6%	40.4%	818
	Inadequate	47.6%	52.4%	1347
Post in root canal (*n* = 12,605)	No post	48.2%	51.8%	10,185
	Fibre post	56.4%	43.6%	165
	Prefabricated post	42.2%	57.8%	1314
	Cast post	54.0%	46.0%	941

^a^Out of the 12 605 endodontically treated teeth, 11 956 teeth (94.8%) were treated using pulpectomy and 649 teeth (5.2%) were treated using pulpotomy method. Root filling length and homogeneity was measured only in pulpectomy group while coronal restoration was assessed in all endodontically treated teeth

Root canal filling (RCF) length and homogeneity were measured only in pulpectomy group while coronal restoration was assessed in all endodontically treated teeth. Shorter RCF than adequate was associated with 1.76 times higher risk for AP while overfilled root canals were associated with 2.51 times higher risk for AP (Table 4). There was 1.61 times higher risk for AP if the RCF was not dense and there were visible voids. There was 2.22 times lower risk for AP in the presence of coronal direct filling compared to no restoration at all (OR = 0.45). There was 2.94 times lower risk for AP if the tooth was restored with crown compared to no restoration at all (OR = 0.34) and 3.03 times lower risk for AP if the tooth was restored with bridge compared to no restoration at all (OR = 0.33). In crowned and bridge retainer teeth also the quality of restoration in terms of gaps or overhangs was evaluated. Inadequate prosthetic restorations increased the risk for AP by 1.63 times while presence of a post in root canal was not a risk factor for AP.

**Table 4:** Associations between clinical characteristics and apical periodontitis (Odds Ratios [OR] and 95% confidence intervals [CI] are presented)

Variables		*P*	OR	95% CI
Length of root filling	0-2 mm from radiographic apex		1.00	
	More than 2 mm short from the radiographic apex	≤0.001	1.76	1.62–1.91
	Extrusion of material through the apex	≤0.001	2.51	2.12–2.97
Density of root filling	Homogenous		1.00	
	Inhomogenous	≤0.001	1.61	1.47–1.76
Restoration	No restoration		1.00	
	Filling	≤0.001	0.45	0.39–0.52
	Crown	≤0.001	0.34	0.29–0.41
	Bridge	≤0.001	0.33	0.27–0.40
Quality of prosthetic restoration	Adequate		1.00	
	Inadequate	0.020	1.63	1.45–1.81
Post in root canal	No post		1.00	
	Fibre post	NS	0.95	0.90–1.24
	Prefabricated	NS	1.68	0.89–2.89
	Cast post	NS	1.34	0.69–2.35
Caries	Absent		1.00	
	Present	≤0.001	2.30	2.14–2.48
Orthodontic appliance	Absent		1.00	
	Present	NS	0.74	0.42–1.34
Tooth localization	Maxilla		1.00	
	Mandibula	≤0.001	2.30	2.14–2.47
Tooth type	Incisor		1.00	
	Canine	0.012	0.79	0.67–0.95
	Premolar	NS	0.97	0.87–1.08
	Molar	0.000	1.79	1.62–1.98
Gender	Female		1.00	
	Male	≤0.001	1.44	1.34–1.55

## Discussion

The authors undertook a cross-sectional observational study involving 6552 patients and focusing on the epidemiology and comorbid factors of periapical radiolucencies in a Southern Estonian population. The study revealed high prevalence of apical periodontitis (AP), observed in 54.7% of the subjects: 10.2% displayed AP without previous RCT or primary apical periodontitis (pAP), 23.9% had only post-treatment AP or secondary apical periodontitis (sAP) and 20.7% of the subjects had both teeth with and without previous RCT (pAP and sAP). A fifth of the 181,495 teeth studied had visible caries and 27.2% had undergone some type of restoration. AP was diagnosed in 6.3% of teeth while 6.9% of teeth were endodontically treated. Only 31% of teeth had received RCF’s of adequate length, 26% were filled homogenously, and only 19% of teeth had RCF’s acceptable in all terms. High quality root canal fillings and presence of coronal restoration were significantly associated with lower risk for AP and therefore better treatment outcome.

Previous endodontic epidemiology studies have displayed numerous variations in study design, investigation methods, sample selection and evaluation criteria. As a result, a direct comparison is clearly difficult, although most of these studies are based on radiographic evaluation. Decades ago, PA radiography was the only imaging method available to diagnose AP [18]. More recently, OPTG radiographs were chosen to assess periapical radiolucency after Muhammed et al. [19] found no statistically significant difference between panoramic radiographs and full-mouth surveys in the detection of periapical radiolucencies. The main advantage of OPTG is that all teeth are visible in a single radiograph; the method also results in relatively lower patient radiation doses. At the same time, this method may not be ideal for the precise analysis of periapical and coronal status of the teeth on the same time [20]. Isolated trabecular bone lesions are difficult to notice in conventional radiographs because of relatively low mineral loss [21]. Overlapping anatomical structures further complicate radiographic diagnosis of AP in two-dimensional images [16]. Therefore, underestimation of AP in OPTG radiographs can be expected [22, 23]. On the other hand, as a limitation of cross-sectional study design, no information is available on the time elapsed between endodontic treatment and the taking of radiographs [24]. All of the healing lesions are thus included in the sCAP group.

The mean age of our study group, 35.5 years, was similar to some other studies [12, 14, 25, 26] but lower than those reported by Tsuneishi et al. [27] (50.8 y), Georgopoulou et al. [28] (48.0 y) and Huumonen et al. [10] (50.2 y). The number of individuals aged between 10 and 19 and 20–29 was significantly higher in our sample compared to other age groups, suggesting that individuals of these age groups tend to seek dental service more frequently. In addition, this age group is more eager to visit a dentist at Tartu University Hospital, since unlike many private clinics, the university hospital has a contract with the Estonian Health Insurance Fund, as well as a separate department of pediatric dentistry. The prevalence of RCF teeth and the presence of AP has been shown to increase with age [15, 27–30]. This is also in line with our findings: the prevalence of AP among teeth without previous endodontic treatment (pAP) was the highest in the 60–69 age group, followed by the 70+ age group. Meanwhile, the prevalence of AP in previously endodontically treated teeth (sAP) was the highest in middle-aged people (50–59 and 40–49 age groups). A similar biased age distribution was also reported in previous studies [31, 32]. The middle-aged population usually possesses better financial possibilities for seeking complex treatment, while older persons, because of their lower socioeconomic status, may be more likely to undergo tooth extraction than root canal treatment.

Our sample contained more females (60.9%) than males (39.1%). Preceding analogous reports have explained emerged gender disparity with women’s higher eagerness in receiving dental care, making them less predisposed to AP [28, 30]. This is furthermore supported by the difference in the prevalence of root fillings in the present study (in 61.0% of women vs. 53.8% of men). Primary AP was also more common in men (33.9%) than in women (28.9%).

The prevalence of AP in our study (54.7%) was higher than generally outlined in other European studies. Higher figs. (63–80%) have only been reported in Belgium, Lithuania, Latvia, Austria, and Belarus in Europe [12–14, 32, 33] while even as much as 87% in Jordan [11]. At the same time, 58.2% of the individuals in our sample had at least one RCT tooth, a result similar to Western European countries – 56.3% in Germany [34], 59% in Spain [35], 61% in Finland [36], and 58.8% in Denmark [37]; while studies conducted in neighbouring Baltic countries (Latvia and Lithuania) reported an even higher prevalence of endodontic treatment among 35–44-year-olds (87% and 84%, respectively) [12, 14]. In the present study, 51.9% of the teeth that had had RCT also displayed AP. This figure was approximating prior reports [27, 38].

Out of all teeth evaluated in our study, 6.3% had signs of periradicular radiolucensies. This figure is lower than reported in studies carried out in some other Eastern and Southeast European countries that showed results ranging from 7% in Latvia to 12.3% in Kosovo [12–14, 39, 40]; but still higher than in Western European countries where periapical lesions were detected 2–5.2% of teeth [35, 37, 41–44].

Some recent studies have shown molars and premolars to be the most common RCF teeth [27, 28] with associated AP [29, 41, 45]. Similar results were reported in the present study, with lower molars and upper premolars requiring the most frequent treatment, followed by the upper molars and incisors. Permanent molar teeth erupt firstly in mouth, therefore they are the most threatened by caries and following pulpitis and AP. They also possess the most complex root canal anatomy [46]. In addition, mandibular molars were often extracted in our study group – lower mandibular first molar was missing in 28.7% of patients. Molar teeth are infrequently needed for aesthetic reasons; in addition to the reasons listed above, smaller efforts for keeping them healthy compared to anterior teeth might therefore play a role in tooth loss [38]. Lastly, the maxillary incisors are most commonly impaired by trauma that is also associated with apical radiolucencies [47].

Dental caries is considered the main cause for pulpal and therefore periodontial disease [48]. Although radiographs often fail to disclose carious lesions [21], our findings verified the existence of a definite association between carious and periapical radiolucencies distinguishable in OPTG-s. Expenses on root canal treatment can be reduced with lowering the prevalence of caries in the general population. Along with this, the sample also reflected the socioeconomic status of our study group: lower income has been reported among Southern Estonian than Northern Estonian population, and overall average income in Estonia is significantly lower than in Western Europe. This population is likely to opt for tooth extraction over root canal treatment followed by costly prosthetic treatment. Keeping in mind the preceding, dental health gives us an overview of the social class, living conditions and general health of the patients, although these variables only have an indirect effect on the occurrence of apical periodontitis. Other studies have demonstrated that regular dental care and caries experiences are strong determinants of periapical disease [29].

Radiographic evidence of lower quality RCF’s contribute to an increase in AP [49]. Moreover, root canal filling per se is the most important risk indicator for AP [50]. There are lot of studies supporting this discovery [15, 28, 30–32, 41, 49, 51–54]. In the current study, more than 50% of RCF teeth were associated with periradicular radiolucencies. This lower-level result, although unexpected, is in line with some previous studies from different countries – high incidence of periradicular lesions in RCF teeth (50% to 62%) has been reported in Brazil [55], Senegal [56], Croatia [40], Palestine [57], and Cyprus [58]. Root canal treatment quality is considered a key factor for the health of periradicular tissues [17, 59]. A recent study by Ng et al. [60] demonstrated the need to improve the technical quality of root canal treatment, especially in molars, to improve periapical health of RCF teeth. Concerning the length of RCF, a meta-analytic study by Kojima et al. [61] reported a significant difference in success rates between under- and overfilled root canal fillings. They concluded that the length of the RCF should be within 2 mm of the radiographic apex. The results of the present study confirmed findings from other studies [32, 55, 59, 62] showing that the apical level of the RCF is strongly associated with periradicular status. In cases where the length of the root filling was 0–2 mm short of the apex, 61% of the teeth showed no periradicular lesion, whereas for underfillings and overfillings, the success rate of the treatment was only 44% and 39%, respectively. The overall high prevalence of AP in our study was certainly associated with the high frequency of inadequate endodontic treatments – of the 11,956 RCF teeth examined in this study, only 31% had RCF of adequate length, only 26% were filled homogenously, and only 19% had RCF acceptable in all terms. Thus, the short length of RCF, extrusion of material through the apex and its presence only in the pulp chamber considerably increased the relative risk for AP. These poor rates indicate a need for improvement of the quality of endodontic treatment in the study region.

At the same time, 40% of the teeth with technically adequate RCF still showed periradicular radiolucencies. Even though some of these lesions might have been actually healing at the time of evaluation, this result still indicates that the quality of RCF is not the only determinant for periradicular status. The presence of microorganisms in the root canal at the time of RCF or later contamination has been shown to increase the risk of failed treatment [63–65]. The quality of the seal created by the coronal restorations is among the factors clearly correlated with the periradicular radiolucencies [14, 29, 55, 66]. Coronal restoration together with the RCF has been suggested to serve as a barrier against bacterial penetration into the periapical area. Moreover, Ray & Trope [67] showed the quality of coronal restoration to be even more important for periapical health than the quality of RCT. Our study indicated that the type of restoration could be a relating factor for periapical lesions. Most of the endodontically treated teeth were restored with a filling, which according to the results is not the best method for preventing AP. The study showed that teeth with indirect coronal restorations demonstrated significantly lower rates of AP compared to direct filling type of restorations while absence of any type of restoration was most commonly associated with AP. The latter is also in line with the results reported by Siquiera et al. [55]. In prosthetic restorations we evaluated also the quality of marginal seal. Marginal gap or overhang was associated with increased prevalence of AP. It must be noted, however, that the true quality of a coronal restoration, presence of small secondary carious lesions, as well as exact cause of AP cannot be identified accurately from a panoramic radiograph. Furthermore, the latter is not sufficient to evaluate the technical procedures and the disinfection protocol used during root canal treatment prior to RCF [64].

Alike Moreno et al. [68] we found no significant associations between presence of root canal posts and of AP. However, this is inconsistent with some other previous epidemiologic studies [69–71] that indicated strong correlation between root canal posts and greater incidence of AP. Increased focus on this parameter is therefore advisable in further studies.

Long-term excessive orthodontic forces have been asserted to cause a predisposition for pulp inflammation and subsequent development of irreversible pulpitis and necrosis [72]. Potential inflammatory and degenerative changes in the pulp of teeth with completed apical formation could be influenced by previous or evolving complications, such as caries or trauma [73]. Furthermore, the orthodontic forces applied to root canal treated teeth do not affect biofilm nor the virulence of microbiota in root canal. To that end, inflammatory periapical lesions should be interpreted to result from the limitations of endodontic treatment [74]. The experimental study on dogs carried out by de Souza et al. [75] indicated that orthodontic movement of teeth with chronic periapical lesion delayed the healing process but did not prevent the periapical lesion to heal. In our study, the presence of fixed orthodontic appliance did not increase the odds ratio for AP. Presumably, orthodontic appliances are more often worn by younger people among whom AP is in general less prevalent. Subjects up to the age of 29 years made up 45.5% of our study group, contributing therefore greatly to this finding.

AP tends to be a common sequela of pulp infection [76] and the local inflammatory process aims to confine and limit the spread of infectious elements [77]. Pulpal and periapical infection can potentially spread throughout the body but the relationship between endodontic inflammation and systemic health has not been meticulously studied. Available scientific evidence suggests that AP may contribute to systemic immune response and systemic inflammation [78–81]. Oxidative stress related to inflammation also has systemic implications, such as increase in cardiovascular and neurodegenerative morbidity [82–85]. On the other hand, surgical endodontic treatment has been shown to reduce systemic inflammation [86]. These studies highlight the relevance of AP to general health, as well as the need for more research on the epidemiology, etiology, and risk factors of this important oral condition.

Our study possesses several strengths. It included 6552 patients, making it one of the most extensive in the field of endodontic epidemiology. The sample size comprised about 0.5% of the total population in Estonia and about 7% of the population of Tartu area. The only recent study to involve a sample of comparable size was carried out in Finland by Huumonen et al. [10] forming much smaller percentage of the total population of Finland or even Helsinki area. In addition, our study included analysis of both teeth and patients, the latter being considerably infrequent in previous studies. Moreover, the study described both endodontically treated and untreated teeth, while also indicating the proportion of intact teeth and other ancillary factors. As a limitation of our study, the sample population described herein only represents the Southern Estonian population treated at the Stomatology Clinic of Tartu University Hospital, and is not representative of the entire Estonian population. Therefore, comparison of current results with other populations should be done cautiously. Dissimilarities in health care system, age, and research methodology will implicate distinctness between the other studies [28, 30, 87].

## Conclusions

Apical periodontitis is highly prevalent in the Southern Estonian population. Most periradicular radiolucencies are associated with root canal-treated teeth, and a half of all root canal-treated teeth show radiological signs of apical periodontitis. The overall quality of root canal fillings tends to be low, consistent with the mediocre outcome of treatment. Considerable efforts are required to improve the standards of endodontic treatment.

## Additional file

Additional file 1: Tables S1-S6. Table S1. Prevalence of apical periodontitis and root canal treatment according to previous studies. **Table S2.** Quality of treatment in case of apical periodontitis according to previous studies. **Table S3.** Distribution of study subjects by age and prevalence of pAP and sAP. **Table S4.** Distribution of teeth in particular tooth groups. **Table S5.** Distribution of teeth with AP and RCT according to tooth FDI number. **Table S6.** Evaluation criteria for radiographic analysis. (DOC 331 kb)

## Abbreviations

AP: Apical periodontitis
OPTG: Orthopantomogram
OR: Odds ratio
pAP: Primary apical periodontitis
RCF: Root canal filling
RCT: Root canal treatment
sAP: Secondary apical periodontitis
