# Supplementary material for: Apical periodontitis in southern Estonian population: prevalence and associations with quality of root canal fillings and coronal restorations
Source: BMC Oral Health. 2017 Dec 12;17:147. doi: 10.1186/s12903-017-0429-7 (PMC5727925; doi:10.1186/s12903-017-0429-7)
Supplement: Additional file 1: Tables S1-S6. Table S1. — Prevalence of apical periodontitis and root canal treatment according to previous studies. Table S2. Quality of treatment in case of apical periodontitis according to previous studies. Table S3. Distribution of study subjects by age and prevalence of pAP and sAP. Table S4. Distribution of teeth in particular tooth groups. Table S5. Distribution of teeth with AP and RCT according to tooth FDI number. Table S6. Evaluation criteria for radiographic analysis. (DOC 331 kb) [file 12903_2017_429_MOESM1_ESM.doc]

**Table S1.** Prevalence of apical periodontitis and root canal treatment according to previous studies

| **Country** | **Total no of teeth** | **Total no of subjects** | **RCT teeth** | **AP teeth** (pAP) | **RCT+AP teeth**  (sAP) | **% of**  **subjects having RCT** | **% of subjects having AP** | **Article** |
| --- | --- | --- | --- | --- | --- | --- | --- | --- |
| **Sweden^1^** | 17430 | 743 | 8,6% | 2,9% | 24,5% | - | 43,2% | Ödesjö et al 1990 |
| **Swizerland^2^** | 2004 | 143 | 20,3% | 8,4% | 31% | 78% | - | Imfeld 1991 |
| **Netherland^3^** | 4196 | 184 | 2,3% | 6% | 39,2% | - | 44,6% | De Cleen et al 1993 |
| **Germany^4^** | 7897 | 323 | 2,7% | 3,0% | 61% | 56,3% |  | Weiger et al 1997 |
| **Lithuania^5^** | 3892 | 147 | 15% | 7,2% | 39,4% | 84% | 70% | Sidaravicius et al 1999 |
| **Belgium^6^** | 4617 | 206 | 6,8% | 6,6% | 40,4% | - | 63,1% | De Moor et al 2000 |
| **Denmark 97/98^7^** | 15 984 | 614 | 4,8% | 3,4% | 52,2% | 52% | 42,3% | Kirkevang et al 2001 |
| **France^8^** | 7561 | 344 | 18,9% | 7,3% | 31,5% |  |  | Lupi-Pegurier et al 2002 |
| **Canada**  **(Toronto)^9^** | 10 474 | 400 | 3,0% | 2,3% untreated with AP | 44,3% | 39% | 32% untreated with AP | Duglas et al 2003 |
| **Canada (Saskatoon)^9^** | 5 674 | 210 | 1.7% | 1,5% untreated with AP | 50,7% | 51% | 21% untreated with AP |
| **Spain # ^10^** | 93 RCT | 180 | 100% | - | 64,5% | - | - | Segura-Egea et al 2004 |
| **Ireland^11^** | 7424 | 302 | 2% | 2% | 25% | 31,8% | 33,1% | Loftus et al 2005 |
| **Belarus^12^** | 31 212 | 1423 | 20% | 12% | 45% | - | 80,2% | Kabak & Abott 2005 |
| **Brasil #^13^** | 2,051RCT | 1139 | 100% | - | 50,3% | - | - | Siqueira et al 2005 |
| **Denmark**  **97/98^14^** | 12 442 | 473 | 4,9% | 3% | 49% | 52,8% | 41,2% | Kirkevang et al 2006 |
| **Denmark**  **2003^14^** | 12 329 | 473 | 5,6% | 3,7% | 44,3% | 58,8% | 50,5% |
| **Turkey^15^** | 8863 | 375 | 5,3% | 4,2% | 53,3% | - | 37,6% | Sunay et al 2007 |
| **Turkey #^16^** | RCT 1268 | RCT 754 | 100% | 40,5% | 40,5% | - | - | Kayahan et al 2008 |
| **Senegal^17^** | 6234 | 208 | 2,6% | 4.6% | 56.1%of roots | 35.5% | 59.6% | Touré et al 2008 |
| **Sweden, Jönköping^18^** | 12433 | 491 | 5,5% | - | 24,4% | - | - | Frisk et al 2008 |
| **Australia^19^** | 5647 | 243 | 8.84% | - | 21.43% | - | - | Da Silva et al 2009 |
| **Iran^20^** | 28463 | 1064 | 3,56% | - | 52% | 41,4% | - | Asgary et al 2010 |
| **Germany^21^** | 4539 | 200 | 4,8% | 5,2% | 38,7% | - | - | Heppeler & Hülsmann 2010 |
| **Italia^22^** | 9423 | 384 | 11,4% | - | 41,6% | - | - | Covello et al 2010 |
| **South-Korea #^23^** | 896 RCT | 742 | 100% | - | 22,8% | 57,7% | - | Kim 2010 |
| **Jordan^24^** | 7,390 | 294 | 5,7% | 11,6% | 71,9% | 63,3% | 83,7% | Al-Omari et al 2011 |
| **Netherland^25^** | 4594 | 178 | 4,8% | 7% | 24,1% | - | 52,2%** | Peters et al 2011 |
| **Turkey^26^** | 28974 | 1077 | 1.6% | 2,2% | 42.0% | 24,4% | - | Gumru et al 2011 |
| **Kosovo^27^** | 4131 | 193 | 2,3% | 12,3% | 46,3% | - | - | Kamberi et al 2011 |
| **Croatia^28^** | 412 | 163 | - | - | 47,3% | - | 47,3% | Peršić et al 2011 |
| **Austria^28^** | 430 | 101 | - | - | 62,1% | - | 62,1% |
| **Croatia^29^** | 38440 | 1462 | 8,5% | 8,5% | 54% | 75,9% | - | Matijevic et al 2011 |
| **Spain,**  **Barcelona^30^** | 9390 | 397 | 6,4% | 2,8% | 23,8% | 59% | 34% | Lopez-Lopez et al 2012 |
| **Finland^31^** | 120 250 | 5244 | 7% | - | - | 61% | - | Huumonen et al 2012 |
| **Creece #^32^** | 469 RCT | 1781 | 100% | - | 62.3% | 17,9% | - | Tolias et al 2012 |
| **Palestine^33^** | 6482 | 258 | 13,2% | 15,1% | 59,5% | 82,2% | - | Mukhaimer et al 2012 |
| **Cypros^34^** | 24,730 | 1006 | 8.9% | 5,5% | 62% | 64% | 68% | Kalender et al 2013 |
| **Turkey^35^** | 23,268 |  | 2,6% | 1,2% | 15,8% |  | - | Ureyen Kaya et al 2013 |
| **Columbia #^36^** | 1086 RCT | 688 | 100% | - | 49% | - | - | Moreno et al 2013 |
| **Latvia^37^** | 7065 | 312 | 18% | 7% | 31-49% * | 87% | 72% | Jersa & Kundzina 2013 |
| **Great Britain^38^** | 3,396 | 136 | 3,4% | 4,1% | 38,3% | - | 49% | Di Filippo et al 2014 |
| **Brazil^39^** | 25,292 | 1,126 | 6,9% | 7.87% | 16,7% | - | - | Berlinck et al 2015 |
| **Finland^40^** | 120635 | 5335 | 6,6% | - | - | - | 27% | Huumonen et al 2017 |
| **Estonia** | 181 495 | 6552 | 6,9% | 6,3% | 51,9% | 58,2% | 54,7% | Present study |

* 31% pulpectomy, 49% pulpotomy

** widening of PDL

# Only the RCT teeth were evaluated.

**Table S2.** Quality of treatment in case of apical periodontitis according to previous studies

| **Country** | **RCT**  **teeth** | **Adequate length** | **Adequate density** | **Adequate length + adequate density** | **adequate RCT+AP** | **inadequate RCT+AP** | **Info about coronal restau-ration** | **Article** |
| --- | --- | --- | --- | --- | --- | --- | --- | --- |
| **Sweden^1^** | 1492 | 41,4% | 30,2% | 15,9% | 17,4% | 21,7% | - | Ödesjö et al 1990 |
| **Switzerland^2^** | 406 | - | - | 36% | - | 48% | - | Imfeld 1991 |
| **Netherland^3^** | 97 | 49,4%- | - | - | 33,3% | 44,9% | + | De Cleen et al 1993 |
| **Germany^4^** | 215 | 41,4% | 33% | 14% | - | - | + | Weiger et al 1997 |
| **Lithuania^5^** | 320 rootfilled  +266 pulpotomy | 33,8% | 30,9% | 37% | 20% | Short +AP 5-30%  Bad obturation +AP 24-30%  Overfilled +AP  66-72% | + | Sidaravicius et al 1999 |
| **Belgium^6^** | 312 | 40,7% | - | - | 25,2% | 53,1% | + | De Moor et al 2000 |
| **Denmark 97/98^7^** | 753 | 54% | 44,1% | - | - | - | - | Kirkevang 2001 |
| **France^8^** | 1429 | 38.7% | 58.9% | 31.2% | 3.8% | 45.3% | - | Lupi-Pegurier et al 2002 |
| **Canada^9^** | 383 | 58,0% | 60,1% | 38.9% | 29,5% | 55,6% | + | Duglas et al 2003 |
| **Spain^10^** | 93 | 52,7% | 50,5% | 34.4% | 46.9% | 75,9% | + | Segura-Egea et al 2004 |
| **Ireland^11^** | 152 | - | - | 47,4% | 86,1% | 65% | + | Loftus et al 2005 |
| **Belarus^12^** | 6339 | 47.7% | - | - | 26,8% | Short+AP 39,3%  Long+ AP 14,5% | + | Kabak & Abott 2005 |
| **Brazil^13^** | 2051 | 59.7% | - | 56.9% | 34,6% | 70,9% | + | Siqueira et al 2005 |
| **Turkey^15^** | 470 | 41,9% | - | - | 11,7% | 83,5% | - | Sunay et al 2007 |
| **Turkey^16^** | 1268 | - | - | 70% | 24,4% | 60,4% | + | Kayahan et al 2008 |
| **Senegal*^17^** | 165 teeth  344 roots* | 22,1% | 7,2% | 17.7% | 26.2% | 62.5% | + | Touré et al 2008 |
| **Sweden**  **Jönköping^18^** | 3981 | 35,8% | 64,8% | 27,4% | 11,8% | 22,8% | - | Frisk et al 2008 |
| **Australia^19^** | 499 | 29.9% | 92.0% | 29.1% | 19,3% | Short+AP 14%  Long+AP 97,14%  Bad density+AP 32,5% | - | Da Silva et al 2009 |
| **Iran^20^** | 1013 | 52,8% | 53,6% | 42.3% | 29.1% | 68,8% | + | Asgary et al 2010 |
| **Germany^21^** | 217 | 45,2% | 54,4% | - | - | - | - | Heppeler et Hülsmann 2010 |
| **Italia^22^** | 1076 | 40,5% | 40,5% | - | Length +AP 28,4%  Density+AP 31,7% | Short +AP 51,8%  Long+AP 30,6%  Bad density+AP  48,4% | + | Covello et al |
| **South-Korea^23^** | 896 | 72,5% | 59,6% | 51,5% | <9% | 48,6% | + | Kim 2010 |
| **Jordan^24^** | 424 | 27.6% | - | - | 32% | 87% | + | Al-Omari et al 2011 |
| **Netherland^25^** | 224 | 44,2% | - | - | 40,4% | 65,6% | + | Peters et al 2011 |
| **Turkey^26^** | 459 | 57,3% | 50,5% | 39,9% | 4,4% | 67.0% | - | Gumru et al 2011 |
| **Kosovo^27^** | 95 | - | - | 30,5% | 20,7% | 57,6% | - | Kamberi et al 2011 |
| **Croatia^28^** | - | 45,6% | - | - | 30,9% | Short+AP 60,7%  Long+AP 70% | - | Peršić et al 2011 |
| **Austria^28^** | - | 50,9% | - | - | 58% | Short+AP 65%  Long +AP 90,9% | - |
| **Croatia^29^** | 3279 | 34,2% | 36,2% | - | - | - | + | Matijevic et al 2011 |
| **Finland^31^** | 8796 | 47% | - | - | - | - | - | Huumonen et al 2012 |
| **Greece^32^** | 469 | 75,1% | 50,2% | - | Good length+AP 54.7 %  Good seal +AP 47.2% | Bad length +AP 79,9%  Bad seal +AP 75,7% | + | Tolias et al 2012 |
| **Palestine^33^** | 855 | 25,5% | - | - | 7,3% | 77,2% | - | Mukhaimer et al 2012 |
| **Cyprus^34^** | 220 | - | - | 38,2% | 26,6% | 87,7% | + | Kalender et al 2013 |
| **Turkey^35^** | 601 | 53,4% | 46,9% | 41,1% | 9,3% | - | + | Ureyen Kaya et al 2013 |
| **Columbia^36^** | 1086 | 52% | - | 33% | 39% | 64% | + | Moreno et al 2013 |
| **Latvia^37^** | 1255 | - | - | 23% | 15% | 35% | - | Jersa & Kundzina 2013 |
| **Great Britain^38^** | 115 | - | - | 55.7% | 14% | 68,6% | - | Di Filippo et al 2014 |
| **Finland^40^** | 7986 | 52% | - | - | 10% | 21% | - | Huumonen et al 2017 |
| **Estonia** | 12 605 | 31.0% | 25.9% | 19.1% | Good length+AP  39.1%;  good density+AP  41.9%;  good length and density +AP  40.0% | Short+AP 56,5%;  overfilled+AP 61,5%;  bad density+AP 51,6% | + | Present study |

* 344 roots were evaluated in 165 teeth, quality was assessed for each root.

**References for Tables S1 and S2.**

^1^Ödesjo B, Hellden L, Salonen L, Langeland K. Prevalence of previous endodontic treatment, technical standard and occurrence of periapieal lesions in a randomly selected adult, general population. Endod Dent Traumatol 1990; 6: 265-272.

^2^Imfeld TN. Prevalence and quality of endodontic treatment in an elderly urban population of Switzerland. J Endod. 1991 Dec;17(12):604-7.

^3^De Cleen MJ, Schuurs AH, Wesselink PR, Wu MK. Periapical status and prevalence of endodontic treatment in an adult Dutch population. Int Endod J. 1993 Mar;26(2):112-9.

^4^Weiger R, Hitzler S, Hermle G, Löst C. Periapical status, quality of root canal fillings and estimated endodontic treatment needs in an urban German population. Endod Dent Traumatol. 1997 Apr;13(2):69-74.

^5^Sidaravicius B, Aleksejuniene J, Eriksen HM. Endodontic treatment and prevalence of apical periodontitis in an adult population of Vilnius, Lithuania. Endod Dent Traumatol. 1999 Oct;15(5):210-5.

^6^De Moor RJG, Hommez GMG, De Boever JG, Delme KIM, Martens GEI. Periapical health related to the quality of root canal treatment in a Belgian population. Int Endod J. 2000 Mar;33(2):113-20.

^7^Kirkevang LL, Horsted-Bindslev P, Ørstavik D, Wenzel A. Frequency and distribution of endodontically treated teeth and apical periodontitis in an urban Danish population. Int Endod J. 2001 Apr;34(3):198-205.

^8^Lupi-Pegurier L, Bertrand MF, Muller-Bolla M, Rocca JP, Bolla M. Periapical status, prevalence and quality of endodontic treatment in an adult French population. Int Endod J. 2002 Aug;35(8):690-7.

^9^Dugas NN, Lawrence HP, Teplitsky PE, Pharoah MJ, Friedman S. Periapical health and treatment quality assessment of root-filled teeth in two Canadian populations. Int Endod J. 2003 Mar;36(3):181-92.

^10^Segura-Egea J, Castellanos-Cosano L, Machuca G, Lopez-Lopez J, Martin-Gonzalez J, Velasco-Ortega E, et al. Diabetes mellitus, periapical inflammation and endodontic treatment outcome. Med Oral Patol Oral Cir Bucal. 2012 Mar 1;17(2):e356-61.

^11^Loftus JJ, Keating AP, McCartan BE. Periapical status and quality of endodontic treatment in an adult Irish population. Int Endod J. 2005 Feb;38(2):81-6.

^12^Kabak Y, Abbott PV. Prevalence of apical periodontitis and the quality of endodontic treatment in an adult Belarusian population. Int Endod J. 2005 Apr;38(4):238-45.

^13^Siqueira JF Jr, Rôças IN, Alves FRF, Campos LC. Periradicular status related to the quality of coronal restorations and root canal fillings in Brazilian population. Oral Surg Oral Med Oral Pathol Oral Radiol Endod. 2005 Sep;100(3):369-74.

^14^Kirkevang LL, Vaeth M, Hörsted-Bindslev P, Wenzel A. Longitudinal study of periapical and endodontic status in a Danish population. Int Endod J. 2006 Feb;39(2):100-7.

^15^Sunay H, Tanalp J, Dikbas I, Bayirli G. Cross-sectional evaluation of the periapical status and quality of root canal treatment in a selected population of urban Turkish adults. Int Endod J. 2007 Feb;40(2):139-45.

^16^Kayahan MB, Malkondu O, Canpolat C, Kaptan F, Bayirli G, Kazazoglu E. Periapical health related to the type of coronal restorations and quality of root canal fillings in a Turkish subpopulation. Oral Surg Oral Med Oral Pathol Oral Radiol Endod. 2008 Jan;105(1):e58-62.

^17^Touré B, Kane AW, Sarr M, Ngom CTH, Boucher Y. Prevalence and technical quality of root fillings in Dakar, Senegal. Int Endod J. 2008 Jan;41(1):41-9.

^18^Frisk F, Hugoson A, Hakeberg M. Technical quality of root fillings and periapical status in root filled teeth in Jönköping, Sweden. Int Endod J. 2008 Nov;41(11):958-68.

^19^Da Silva K, Lam JMY, Wu N, Duckmanton P. Cross-sectional study of endodontic treatment in an Australian population. Aust Endod J. 2009 Dec; 35(3): 140–146.

^20^Asgary S, Shadman B, Ghalamkarpour Z, Shahravan A, Ghoddusi J, Bagherpour A, Akbarzadeh Baghban A, Hashemipour M, Ghasemian Pour M. Periapical Status and Quality of Root canal Fillings and Coronal Restorations in Iranian Population. Iran Endod J. 2010 Spring;5(2):74-82.

^21^Heppeler J, Hülsmann M. Prevalence of root canal fillings, apical periodontitis, and endodontic treatment needs in a selected German population in 1994 and 2004. ENDO (Lond Engl) 2010;4(3):189–200.

^22^Covello F, Franco V, Schiavetti R, Clementini M, Mannocci A, Ottria L, Costacurta M. Prevalence of apical periodontitis and quality of endodontic treatment in an Italian adult population. Oral Implantol (Rome). 2010 Oct-Dec; 3(4): 9–14.

^23^Kim S. Prevalence of apical periodontitis of root canal-treated teeth and retrospective evaluation of symptom-related prognostic factors in an urban South Korean population. Oral Surg Oral Med Oral Pathol Oral Radiol Endod. 2010 Dec;110(6):795-9.

^24^Al-Omari MA, Hazaa A, Haddad F. Frequency and distribution of root filled teeth and apical periodontitis in a Jordanian subpopulation. Oral Surg Oral Med Oral Pathol Oral Radiol Endod. 2011 Jan;111(1):e59-65.

^25^Peters LB, Lindeboom JA, Elst ME, Wesselink PR. Prevalence of apical periodontitis relative to endodontic treatment in an adult Dutch population: a repeated cross-sectional study. Oral Surg Oral Med Oral Pathol Oral Radiol Endod. 2011 Apr;111(4):523-8.

^26^Gumru B, Tarcin B, Pekiner FN, Ozbayrak S. Retrospective radiological assessment of root canal treatment in young permanent dentition in a Turkish subpopulation. Int Endod J. 2011 Sep;44(9):850-6.

^27^Kamberi B, Hoxha V, Stavileci M, Dragusha E, Kuci A, Kqiku L. Prevalence of apical periodontitis and endodontic treatment in a kosovar adult population. BMC Oral Health. 2011 Nov 29;11:32.

^28^Peršić R, Kqiku L, Brumini G, Husetić M, Pezelj-Ribarić S, Brekalo Pršo I, Städtler P. Difference in the periapical status of endodontically treated teeth between the samples of Croatian and Austrian adult patients. Croat Med J. 2011 Dec 15;52(6):672-8.

^29^Matijević J, Cizmeković Dadić T, Prpic Mehicic G, Ani I, Slaj M, Jukić Krmek S. Prevalence of apical periodontitis and quality of root canal fillings in population of Zagreb, Croatia: a cross-sectional study. Croat Med J. 2011 Dec 15;52(6):679-87.

^30^López-López J, Jané-Salas E, Estrugo-Devesa A, Castellanos-Cosano L, Martín-González J, Velasco-Ortega E, Segura-Egea JJ. Frequency and distribution of root-filled teeth and apical periodontitis in an adult population of Barcelona, Spain. Int Dent J. 2012 Feb;62(1):40-6.

^31^Huumonen S, Vehkalahti MM, Nordblad A. Radiographic assessments on prevalence and technical quality of endodontically-treated teeth in the Finnish population, aged 30 years and older. Acta Odontol Scand. 2012 May;70(3):234-40.

^32^Tolias D, Koletsi K, Mamai-Homata E, Margaritis V, Kontakiotis E. Apical periodontitis in association with the quality of root fillings and coronal restorations: a 14-year investigation in young Greek adults. Oral Health Prev Dent. 2012;10(3):297-303.

^33^Mukhaimer R, Hussein E, Orafi I. Prevalence of apical periodontitis and quality of root canal treatment in an adult Palestinian sub-population. Saudi Dent J. 2012 Jul;24(3-4):149-55.

^34^Kalender A, Orhan K, Aksoy U, Basmaci F, Er F, Alankus A. Influence of the quality of endodontic treatment and coronal restorations on the prevalence of apical periodontitis in a Turkish Cypriot population. Med Princ Pract. 2013;22(2):173-7.

^35^Ureyen Kaya B, Kececi AD, Guldas HE, Orhan H. A retrospective radiographic study of coronal-periapical status and root canal filling quality in a selected adult Turkish population. Med Princ Pract. 2013;22(4):334-9.

^36^Moreno JO, Alves FR, Gonçalves LS, Martinez AM, Rôças IN, Siqueira JF Jr. Periradicular status and quality of root canal fillings and coronal restorations in an urban Colombian population. J Endod. 2013 May;39(5):600-4.

^37^Jersa I, Kundzina R. Periapical status and quality of root fillings in a selected adult Riga population. Stomatologija. 2013;15(3):73-7.

^38^Di Filippo G, Sidhu SK, Chong BS. Apical periodontitis and the technical quality of root canal treatment in an adult sub-population in London. Br Dent J. 2014 May;216(10):E22.

^39^Berlinck T, Tinoco JM, Carvalho FL, Sassone LM, Tinoco EM. Epidemiological evaluation of apical periodontitis prevalence in an urban Brazilian population. Braz Oral Res. 2015;29:51.

^40^Huumonen S, Suominen AL, Vehkalahti MM. Prevalence of apical periodontitis in root filled teeth: findings from a nationwide survey in Finland. Int Endod J. 2017 Mar;50(3):229-236.

**Table S3.** Distribution of study subjects by age and prevalence of pAP and sAP.

| Study subjects | | | AP wo RCT (pAP) | | AP with RCT (sAP) | |
| --- | --- | --- | --- | --- | --- | --- |
| Age group | No of subjects | Percent | No of subjects with AP in teeth wo RCT (pAP) | Teeth with pAP  per patient  Mean ± SD | No of subjects with AP in RCT teeth (sAP) | Teeth with sAP per patient  Mean ± SD |
| <5 | 9 | 0,1 | 0 | 0,0 ± 0,0 | 0 | 0,0 ± 0,0 |
| 5-9 | 352 | 5,4 | 4 | 0,0 ± 0,2 | 6 | 1,0 ± 0,0 |
| 10-14 | 586 | 8,9 | 18 | 0,0 ± 0,2 | 21 | 1,4 ± 1,2 |
| 15-19 | 740 | 11,3 | 85 | 0,2 ± 0,7 | 99 | 1,5 ± 1,0 |
| 20-24 | 647 | 9,9 | 132 | 0,4 ± 1,1 | 158 | 1,6 ± 0,8 |
| 25-29 | 648 | 9,9 | 163 | 0,6 ± 1,6 | 273 | 1,7 ± 1,1 |
| 30-34 | 530 | 8,1 | 172 | 0,8 ± 1,7 | 267 | 2,0 ± 1,5 |
| 35-39 | 487 | 7,4 | 209 | 1,1 ± 2,0 | 304 | 2,3 ± 1,5 |
| 40-44 | 463 | 7,1 | 219 | 1,1 ± 1,9 | 334 | 2,4 ± 1,6 |
| 45-49 | 427 | 6,5 | 191 | 1,2 ± 2,1 | 300 | 2,5 ± 1,8 |
| 50-54 | 410 | 6,3 | 194 | 1,2 ± 1,8 | 299 | 2,6 ± 1,8 |
| 55-59 | 369 | 5,6 | 168 | 1,1 ± 1,7 | 274 | 2,6 ± 1,8 |
| 60-64 | 280 | 4,3 | 151 | 1,3 ± 1,7 | 206 | 2,5 ± 1,8 |
| 65-69 | 232 | 3,5 | 121 | 1,3 ± 1,9 | 150 | 2,4 ± 1,7 |
| 70-74 | 186 | 2,8 | 97 | 1,2 ± 1,8 | 117 | 2,4 ± 1,8 |
| 75-79 | 101 | 1,5 | 52 | 1,5 ± 2,3 | 62 | 1,9 ± 1,3 |
| 80-84 | 60 | 0,9 | 33 | 1,5 ± 2,1 | 37 | 2,0 ± 1,4 |
| 85-89 | 23 | 0,4 | 11 | 1,2 ± 1,8 | 12 | 2,2 ± 1,2 |
| >=90 | 2 | 0,0 | 1 | 1,5 ± 2,1 | 1 | 1,0 ± 0,0 |
| **Total** | **6552** | **100,0** | **2021** | **0,8 ± 1,6** | **2920** | **2,2 ± 1,6** |

**Table S4.** Distribution of teeth in particular tooth groups

|  | | Total | Teeth Present | | pAP teeth | | P value | RCT teeth | | P value | sAP teeth | | P value |
| --- | --- | --- | --- | --- | --- | --- | --- | --- | --- | --- | --- | --- | --- |
|  |  |  | No of teeth | % | No of teeth | % * |  | No of teeth | % * |  | No of teeth | % # |  |
| Upper teeth | **Total** | **104832** | **90214** | **86.1** | **2011** | **2.2** | <0.001 | **7305** | **8.1** | <0.001 | **3163** | **43.3** | <0.001 |
|  | Molars | 39312 | 30415 | 77.4 | 923 | 3.0 |  | 2656 | 8.7 |  | 1248 | 47.0 |  |
|  | Premolars | 26208 | 22364 | 85.3 | 479 | 2.1 |  | 2376 | 10.6 |  | 949 | 39.9 |  |
|  | Canines | 13104 | 12630 | 96.4 | 160 | 1.3 |  | 500 | 4.0 |  | 189 | 37.8 |  |
|  | Incisors | 26208 | 24805 | 94.6 | 449 | 1.8 |  | 1773 | 7.1 |  | 777 | 43.8 |  |
| Lower teeth | **Total** | **104832** | **91281** | **87.1** | **2887** | **3.2** | <0.001 | **5300** | **5.8** | <0.001 | **3377** | **63.7** | <0.001 |
|  | Molars | 39312 | 28689 | 73.0 | 1904 | 6.6 |  | 3281 | 11.4 |  | 2318 | 70.6 |  |
|  | Premolars | 26208 | 24197 | 92.3 | 600 | 2.5 |  | 1563 | 6.5 |  | 818 | 52.3 |  |
|  | Canines | 13104 | 12933 | 98.7 | 108 | 0.8 |  | 175 | 1.4 |  | 81 | 46.3 |  |
|  | Incisors | 26208 | 25462 | 97.2 | 275 | 1.1 |  | 281 | 1.1 |  | 160 | 56.9 |  |
| Total |  | **209664 &** | **181495**  **¤** | **86.6** | **4898** | **2.7** |  | **12605** | **6.9** |  | **6540** | **51.9** |  |

%* - pAP and RCT percentages are calculated of total number of teeth present

% # - sAP is calculated from RCT teeth

& - maximum nominal number of teeth (32 teeth x 6552 patients).

¤ - number of teeth actually present in 6552 patients (including wisdom teeth).

**Table S5.** Distribution of teeth with AP and RCT according to tooth FDI number

| **FDI**  **Tooth no** | **Total**  **No of**  **teeth** | No of Healthy teeth | **%**  **of healthy teeth** | No of pAP teeth | **% of pAP teeh** | No of RCT teeth | **% of RCT teeth** | No of sAP teeth | **% of sAP teeth (from RCT** |
| --- | --- | --- | --- | --- | --- | --- | --- | --- | --- |
| 11 | 6280 | 5718 | 91,1% | 92 | 1,5% | 470 | 7,5% | 179 | 38% |
| 12 | 6140 | 5652 | 92,1% | 121 | 2,0% | 367 | 6,0% | 169 | 46,0% |
| 13 | 6324 | 5979 | 94,5% | 85 | 1,3% | 260 | 4,1% | 98 | 37,7% |
| 14 | 5714 | 5034 | 88,1% | 134 | 2,3% | 546 | 9,6% | 215 | 39,4% |
| 15 | 5492 | 4642 | 84,5% | 142 | 2,6% | 708 | 12,9% | 277 | 39,1% |
| 16 | 5509 | 4485 | 81,4% | 189 | 3,4% | 835 | 15,2% | 378 | 45,3% |
| 17 | 6317 | 5697 | 90,2% | 181 | 2,9% | 439 | 6,9% | 223 | 50,8% |
| 18 | 3714 | 3527 | 95,0% | 106 | 2,9% | 81 | 2,2% | 40 | 49,4% |
| 21 | 6265 | 5635 | 90,0% | 117 | 1,9% | 513 | 8,2% | 204 | 39,8% |
| 22 | 6120 | 5578 | 91,1% | 119 | 1,9% | 423 | 6,9% | 225 | 53,2% |
| 23 | 6306 | 5991 | 95,0% | 75 | 1,2% | 240 | 3,8% | 91 | 37,9% |
| 24 | 5700 | 5104 | 89,5% | 99 | 1,7% | 497 | 8,7% | 194 | 39,0% |
| 25 | 5458 | 4729 | 86,6% | 104 | 1,9% | 625 | 11,5% | 263 | 42,1% |
| 26 | 5455 | 4489 | 82,3% | 183 | 3,4% | 783 | 14,4% | 349 | 44,6% |
| 27 | 5721 | 5099 | 89,1% | 156 | 2,7% | 466 | 8,1% | 234 | 50,2% |
| 28 | 3699 | 3539 | 95,7% | 108 | 2,9% | 52 | 1,4% | 24 | 46,2% |
| 31 | 6329 | 6197 | 98,0% | 58 | 0,9% | 74 | 1,2% | 41 | 55,4% |
| 32 | 6407 | 6266 | 97,8% | 76 | 1,2% | 65 | 1,0% | 37 | 56,9% |
| 33 | 6474 | 6334 | 97,8% | 53 | 0,8% | 87 | 1,3% | 38 | 43,7% |
| 34 | 6294 | 5945 | 94,5% | 112 | 1,8% | 237 | 3,8% | 122 | 51,5% |
| 35 | 5797 | 5103 | 88,0% | 181 | 3,1% | 513 | 8,8% | 274 | 53,4% |
| 36 | 4670 | 3402 | 72,8% | 362 | 7,8% | 906 | 19,4% | 671 | 74,1% |
| 37 | 5457 | 4523 | 82,9% | 376 | 6,9% | 558 | 10,2% | 366 | 65,6% |
| 38 | 4135 | 3757 | 90,9% | 235 | 5,7% | 143 | 3,5% | 84 | 58,7% |
| 41 | 6331 | 6200 | 97,9% | 60 | 0,9% | 71 | 1,1% | 35 | 49,3% |
| 42 | 6395 | 6243 | 97,6% | 81 | 1,3% | 71 | 1,1% | 47 | 66,2% |
| 43 | 6459 | 6316 | 97,8% | 55 | 0,9% | 88 | 1,4% | 43 | 48,9% |
| 44 | 6299 | 5915 | 93,9% | 121 | 1,9% | 263 | 4,2% | 122 | 46,4% |
| 45 | 5807 | 5071 | 87,3% | 186 | 3,2% | 550 | 9,5% | 300 | 54,5% |
| 46 | 4755 | 3467 | 72,9% | 340 | 7,2% | 948 | 19,9% | 716 | 75,5% |
| 47 | 5502 | 4580 | 83,2% | 340 | 6,2% | 582 | 10,6% | 378 | 64,9% |
| 48 | 4170 | 3775 | 90,5% | 251 | 6,0% | 144 | 3,5% | 103 | 71,5% |
| **Total** | **181 495 ¤** | 163992 | 90,4% | 4898 | 2,7% | 12605 | 6,9% | 6540 | 51,9% |

¤ - number of teeth actually present in 6552 patients (including wisdom teeth).

**Table S6**. Evaluation criteria for radiographic analysis

| Evaluation criteria | Coded- value |
| --- | --- |
| Periapical pathology (PA) | 0 – without PA radiolucency (PAI ≤ 2) |
|  | 1 – visible PA radiolucency (PAI >2) |
| Method of RCT | 0 - pulpotomy |
|  | 1 - pulpectomy |
| RCF length | 0 – adequate (no more than >2mm shorter from radiographic apex) |
|  | 1 –shorter than >2mm from radiographic apex |
|  | 2 – overfilled RCF |
| RCF density | 0 – adequate (dense and homgenous in all aspects of RCF) |
|  | 1 – inadequate (sparse, voids, gaps in RCF or between RCF and RC) |
| Type of restoration | 0 – missing restoration |
|  | 1 - filling |
|  | 2 - crown |
|  | 3 – bridge abutment tooth |
| Presence of caries | 0 – no visible caries |
|  | 1 – primary caries  2 – secondary caries |
| Post in RC | 0 – no post |
|  | 1 – fiber post |
|  | 2 – prefabricated screw or post |
|  | 3 – cast post and core |
| Quality of prosthetic restoration | 0 – adequate (no gap, no overhang) |
|  | 1 – inadequate (gap between core and crown, overhanging crown) |
|  |  |
| We also evaluated:  presence, type and situation of dental implants;  presence, type of orthodontic apliances;  presence, angulation, level of eruption of wisdom teeth  presence, coronal and radicular situations of all decidious teeth | |
